# 657. Short-Course vs. Long-Course Antibiotic Therapy for Uncomplicated Enterococcal Bacteremia

**DOI:** 10.1093/ofid/ofaf695.212

**Published:** 2026-01-11

**Authors:** Mayte Lezcano, Christine A Vu, Jyoti Somani

**Affiliations:** Jackson Memorial Hospital, Miami, FL; Jackson Memorial Hospital, Miami, FL; Jackson Health Systems, Miami, Florida

## Abstract

**Background:**

The optimal duration of treatment for uncomplicated enterococcal bacteremia remains undefined. The 2009 Infectious Diseases Society of America (IDSA) recommends 7 to 14 days of treatment for central-line associated enterococcal bacteremia. We aimed to compare the outcomes of short-course antibiotic therapy (4-10 days) versus long-course therapy (11-17 days) in patients with uncomplicated enterococcal bacteremia.

**Figure:**
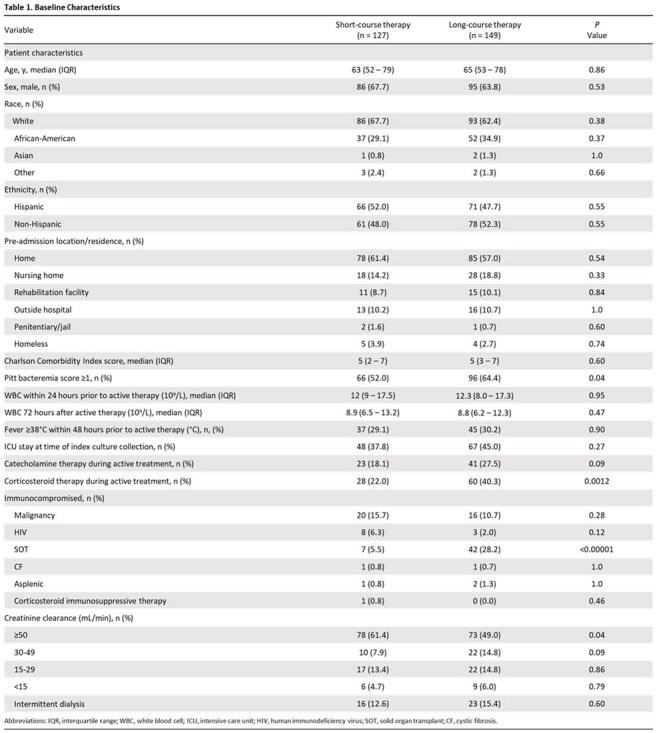

**Figure:**
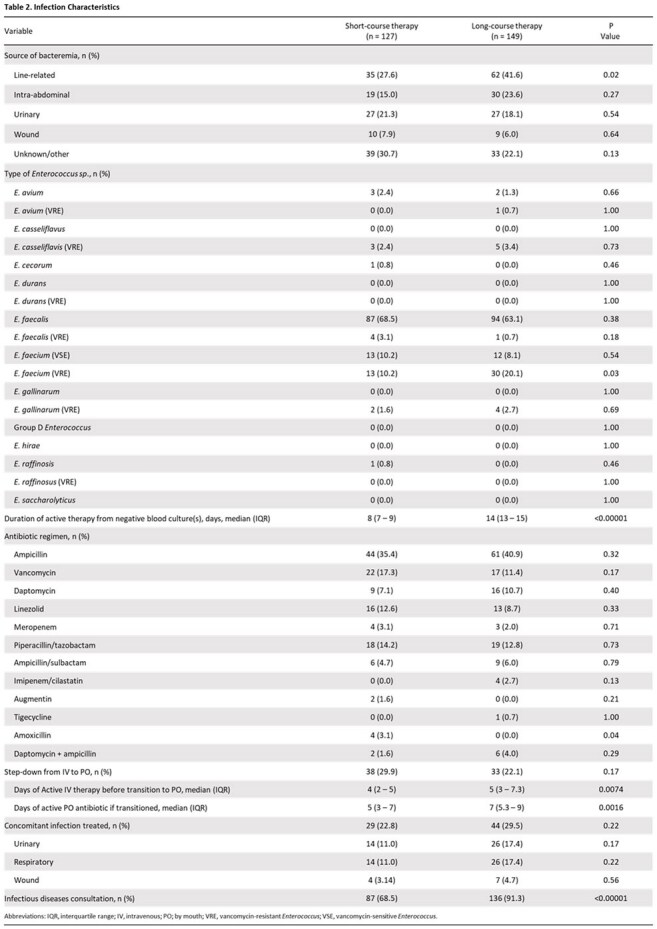

**Methods:**

A retrospective study was conducted on adults treated for uncomplicated enterococcal bacteremia between January 1^st^, 2021 and December 31^st^, 2024 across four acute-care hospitals in Miami, Florida. Uncomplicated enterococcal bacteremia was defined as having a monomicrobial positive blood culture with *Enterococcus sp*., along with clinical stability and microbiological clearance within 72 hours following treatment. Persistent bacteremia, lack of source control, and deep-seated infections were excluded. The primary outcome was 30-day mortality and secondary outcomes included clinical cure, 30-day microbiological relapse, antibiotic-free days, hospital length-of-stay, 90-day *Clostridium difficile* rate, and 30-day hospital readmission rate. A sample size of 276 patients was needed to achieve 80% power and a non-inferiority margin was set at 10%.

**Figure:**
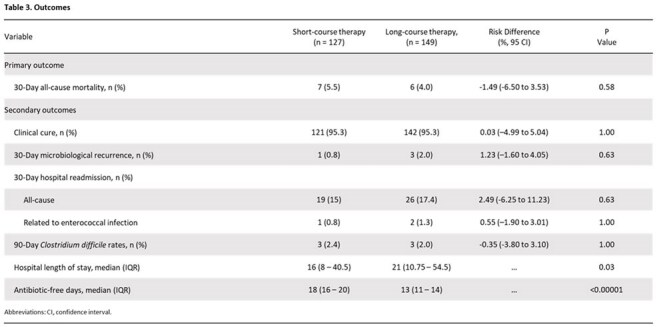

**Results:**

Of the 1,140 patients screened, 276 patients were included: 127 (46%) patients in short-course treatment and 149 (54%) patients in long-course treatment. The primary outcome of 30-day all-cause mortality occurred in 7 patients (5.5%) in the short-course group vs 6 patients (4.0%) in the long-course group ([risk difference, -1.49% [95% confidence interval, –6.50% to 3.53%]). No significant differences were observed in clinical cure, microbiological relapse, hospital readmission, and *Clostridium difficile* infections. Short-course compared to long-course therapy had reduced hospital length of stay (16 vs. 21 days; p< 0.03) and increased antibiotic-free days (18 vs. 13 days; p< 0.00001); step-down from intravenous to oral therapy occurred at a median of 4 and 5 days, respectively.

**Conclusion:**

Among hospitalized patients with uncomplicated enterococcal bacteremia, antibiotic treatment with short-course therapy was noninferior to treatment with long-course therapy.

**Disclosures:**

All Authors: No reported disclosures

